# Accurate Machine Learning Model to Diagnose Chronic Autoimmune Diseases Utilizing Information From B Cells and Monocytes

**DOI:** 10.3389/fimmu.2022.870531

**Published:** 2022-04-20

**Authors:** Yuanchen Ma, Jieying Chen, Tao Wang, Liting Zhang, Xinhao Xu, Yuxuan Qiu, Andy Peng Xiang, Weijun Huang

**Affiliations:** ^1^ Center for Stem Cell Biology and Tissue Engineering, Key Laboratory for Stem Cells and Tissue Engineering, Ministry of Education, Sun Yat-sen University, Guangzhou, China; ^2^ Zhongshan School of Medicine, Sun Yat-sen University, Guangzhou, China

**Keywords:** chronic autoimmune disease, accurate diagnosis, machine learning (ML), scRNA-seq, cellular cross talking

## Abstract

Heterogeneity and limited comprehension of chronic autoimmune disease pathophysiology cause accurate diagnosis a challenging process. With the increasing resources of single-cell sequencing data, a reasonable way could be found to address this issue. In our study, with the use of large-scale public single-cell RNA sequencing (scRNA-seq) data, analysis of dataset integration (3.1 × 10^5^ PBMCs from fifteen SLE patients and eight healthy donors) and cellular cross talking (3.8 × 10^5^ PBMCs from twenty-eight SLE patients and eight healthy donors) were performed to identify the most crucial information characterizing SLE. Our findings revealed that the interactions among the PBMC subpopulations of SLE patients may be weakened under the inflammatory microenvironment, which could result in abnormal emergences or variations in signaling patterns within PBMCs. In particular, the alterations of B cells and monocytes may be the most significant findings. Utilizing this powerful information, an efficient mathematical model of unbiased random forest machine learning was established to distinguish SLE patients from healthy donors *via* not only scRNA-seq data but also bulk RNA-seq data. Surprisingly, our mathematical model could also accurately identify patients with rheumatoid arthritis and multiple sclerosis, not just SLE, *via* bulk RNA-seq data (derived from 688 samples). Since the variations in PBMCs should predate the clinical manifestations of these diseases, our machine learning model may be feasible to develop into an efficient tool for accurate diagnosis of chronic autoimmune diseases.

## Introduction

Systemic lupus erythematosus (SLE), multiple sclerosis (MS), and rheumatoid arthritis (RA) are all chronic autoimmune diseases associated with progressive widespread organ damage (1–3). The course of these three diseases is typically progressive with intermittent remission (4, 5). It is generally accepted that early treatment could increase the remission probability of these diseases and improve their prognosis (6, 7). If appropriate treatment is not given in a timely manner, these diseases may progress, causing work disability and life quality reduction for patients. Furthermore, such progression would lead to enormous financial burdens to the patients, their families, and society (8–10). Hence, it is crucial to develop an efficient method of accurate diagnosis to enable early intervention for these diseases.

Unfortunately, it seems that diagnosing SLE, MS, and RA may still be a challenging process that relies on a set of criteria (11–13), including clinical manifestations, functional outcomes, and serological and radiological evidence, that have to be met to make an accurate diagnosis (14, 15). Under non-specific and insensitive criteria, the misdiagnosis and underdiagnosis of these diseases are relatively common (16). The average time from symptom onset to diagnosis confirmation was approximately two years (17). This may cause patients to miss the optimal time for treatment. To break the bottleneck of early diagnosis, many studies have focused on biomarker detection to develop an accurate diagnostic criterion (18–21). However, the results were unsatisfying, owing to the tremendous heterogeneity of these diseases and limited comprehension of the disease pathophysiology (22).

In detail, although it is well known that the loss of immune tolerance and persistent release of autoantibodies are the two important bases for the pathophysiology of chronic autoimmune disease (23, 24), most studies have focused on investigating the contribution of certain cellular or molecular mechanisms rather than comprehensively and systematically illustrating the pathogenesis. This might be due to the limitation of methods or means. With the development of single-cell sequencing technology, the increased resources of data, and the improvement of bioinformatic tools (e.g., Seurat, SHARP, CellChat, etc.) (25–27), these would together help us to comprehend the pathophysiology of these diseases, thus their crucial features would be efficient for being mined. For example, Nehar-Belaid et al. thoroughly analyzed the major cell types among peripheral blood mononuclear cells and revealed an expanded subpopulation that has a specific interferon-stimulated gene (ISG) expression pattern in SLE patients (28). Meena Subramaniam et al. also found that monocytes from SLE patients highly expressed ISGs (29). Both of these studies comprehensively illuminated the cytological changes of SLEs.

**Figure 1:**
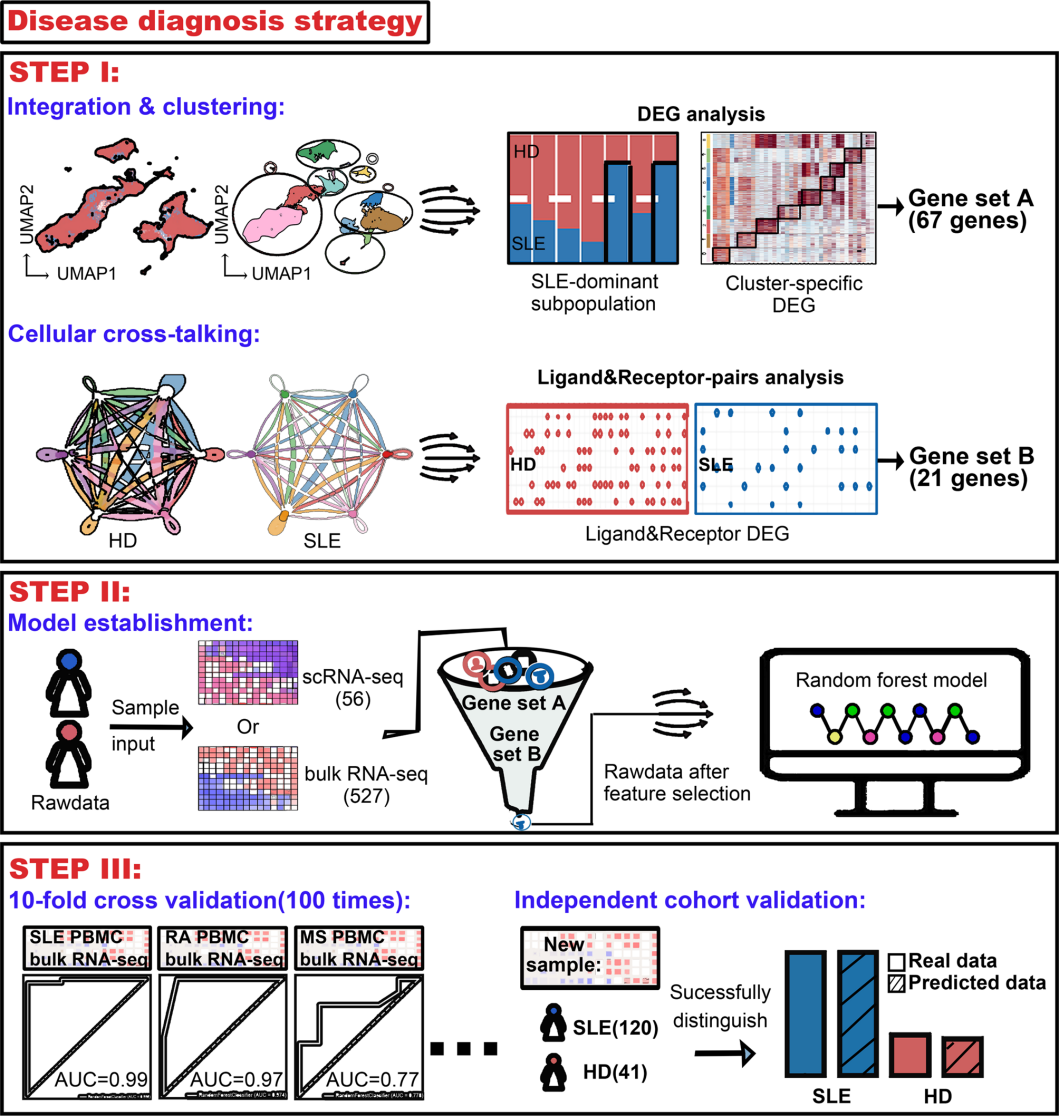
Workflow for establishment of an accurate machine learning model to diagnose chronic autoimmune diseases. STEP I, to figure out the most crucial information that characterizes diseases using public scRNA-seq datasets. From analysis of integration and clustering, 67 top five cluster-specific genes basing on the differential expression gene identification within SLE dominant PBMC subpopulations were derived. From cellular cross-talking analysis, 21 genes constituting ligand–receptor pairs disappeared in SLE patients and showed that more than two kinds of PBMC subpopulation were derived. A union of these two gene sets would be used in the next step. STEP II, to establish the machine learning model diagnosing diseases. A random forest machine learning model was implemented, and genes derived from step I were combined as feature input. 56 and 527 samples were used as sample input for scRNA-seq and bulk RNA-seq data, respectively. STEP III, to validate the accuracy of our machine learning model. Receiver operating characteristic (ROC) analysis was used to test the accuracy, and multiple times of ten-fold cross-validation tests were adopted to avoid bias. The diagnostic accuracy of our model was also validated using an independent bulk RNA-seq cohort containing 120 SLE patients and 41 health donors.

According to these public single-cell RNA sequencing (scRNA-seq) data of SLE, we seek for a feasible way for SLE accurate diagnosis. Firstly, integration and cellular cross-talking analysis were performed to obtain the powerful information labeling the disease. This information was then combined with an unbiased random forestry machine learning algorithm which rendered an efficient mathematical model for SLE diagnosis. The accuracy of the mathematical model to identify patients with RA and MS was also validated. Furthermore, the diagnostic precision of our model was evaluated using an independent SLE cohort (**Figure 1**).

## Material and Methods

### Data Availability

The single-cell RNA sequencing data were deposited in the Gene Expression Omnibus (GEO), and the accession numbers were GSE137029 and GSE135779 for SLE patients and GSE164378 for healthy donors. Bulk RNA-sequencing data were deposited to GSE72509 and GSE164457 for peripheral blood mononuclear cells (PBMCs) of SLE patients, GSE90081 for PBMCs of RA patients, GSE89408 for synovial tissues of RA patients, GSE159225 for PBMCs of MS patients, and GSE89408 for CD14-positive cells of MS patients, and GSE183204 and GSE169687 for PBMCs of healthy donors.

### Integration of Single-Cell RNA Sequencing Data

Reciprocal principal component analysis (RPCA)-based integration could effectively detect a state-specific cell cluster and run significantly faster on large datasets. Compared with other integration tools (e.g., BBKNN and LIGER), RPCA could conserve more distinct cell identities when removing batch effect, particularly for the data of immune cells (30). Considering its balancing capability on batch effect removal and biological variance preserving, RPCA would be used for our dataset integration. Before the integration, two lists were created: one containing merged SLE data and the other containing merged healthy data. These two lists were then combined and integrated through Seurat (version 4.0.5) following the guidelines at https://satijalab.org/seurat/articles/integration_rpca.html.

### PBMCs and Their Subpopulation Clustering

To discover SLE-dominant cell clusters, PBMCs and their subpopulations were clustered through Seurat (version 4.0.5), respectively. Cell proportions of each cluster were calculated subsequently. For PBMC cell clustering, each cell subcluster was annotated based on a canonical marker. Any cluster that has SLE cells containing more than 75% would be considered as SLE dominant.

### Differential Expression Gene Analysis on SLE-Dominant Cell Clusters

Within those PBMC subpopulations (e.g., B cells and monocytes) which contain the SLE-dominated cluster, differential expression gene (DEG) analysis would be applied on all of their cell clusters with Function *FindAllMarkers* embedded in Seurat (version 4.0.5) to find out useful information that mark the SLE state. Top five genes based on their log2 fold change value were selected as the first part of feature input for machine learning. Meanwhile, these DEG functions were annotated through literature search.

### Cellular Cross-Talking Analysis

The machine learning model can be optimized with powerful sources of information. Thus, CellChat (version 1.1.3) analysis was performed following the guidelines at https://github.com/sqjin/CellChat. In details, overall interaction, overall signaling pattern, outgoing/incoming signaling pattern, and ligand–receptor pair were checked step by step. Samples were analyzed independently. Datasets of patients and health donors were analyzed separately and merged to make a comparison analysis. Ligand–receptor pairs which disappeared at SLE were selected as a second part of feature input for machine learning.

### Machine Learning With the Random Forest Model

The random forest machine learning model was implemented with sklearn (version 0.23.2). The gene set which derived from integration and CellChat analysis were combined as feature input, aiming at selecting information within the sequencing datasets, thus improving the performance of the machine learning model. 56 and 527 samples were used as sample input for scRNA-seq and bulk RNA-seq data, respectively. Samples from patients and healthy donors were labeled with 1 and 0, respectively. With the function *train_test_split* within *sklearn.model_selection*, the data were split into two parts, 70% for training and 30% for testing, according to previous study (31). Data balancing was performed when the cell/sample ratio between patients and healthy donors was above 1:2, at random forest model initialization. Receiver operating characteristic (ROC) analysis was used to test models’ accuracy. The models for each disease were independent.

To avoid bias of data composition, the sklearn module *StratifiedKFold* was used to split data into ten parts preserving the ratio of samples and perform a ten-fold cross-validation with a loop of one hundred. The average and standard deviation of area under curve (AUC) were documented.

### Diagnostic Accuracy Validation of the Machine Learning Model

An independent bulk RNA-seq cohort containing 120 SLE patients and 41 health donors was enrolled into the diagnostic accuracy validation of our machine learning model. Basic information of this cohort including SLE severity, age, and gender was documented. Genes which were used as feature input for the machine learning model were confirmed to be expressed in each sample. The diagnostic accuracy of our machine learning model for SLE and healthy donors was tested separately.

### Statistical Analysis

The statistical significance of differential gene expression was analyzed with the Wilcoxon test, a default parameter in function *FindAllMarkers* of Seurat packages.

### Software Version

All the software mentioned above were based on R (version 4.1.1) and Python (3.7). Integration analysis and cell clustering were based on Seurat (version 4.0.5), and cellular cross-talking analysis was based on CellChat (1.1.3). Machine learning was based on sklearn (version 0.23.2).

## Results

### The Limited Alterations of Cell Composition in SLE Patients From the Overall PBMC Perspective

**Figure 2:**
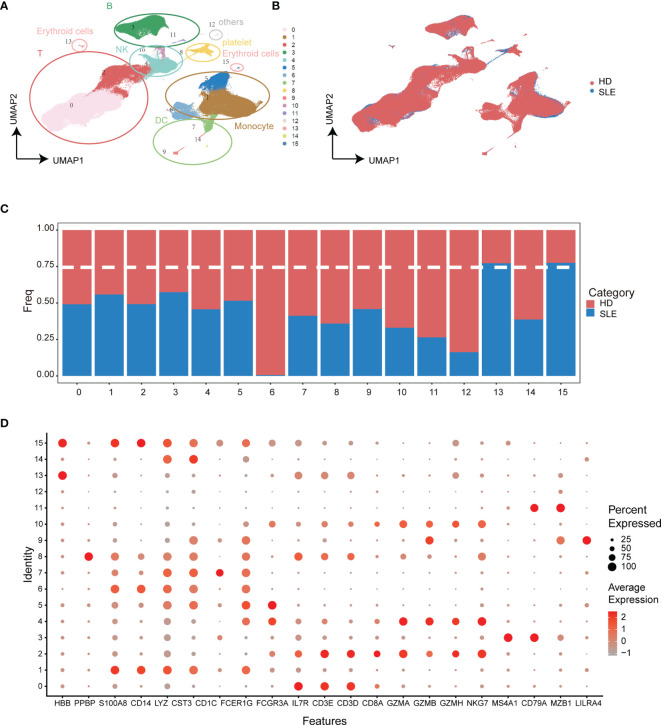
Integration analysis of single-cell RNA sequencing datasets from SLE patients and healthy donors. **(A)** UMAP plot of categorized cell clusters. **(B)** UMAP plot of single-cell PBMCs from fifteen SLE flare patients and eight healthy donors. **(C)** Bar plot of cell proportion in each cell cluster. The dashed line represents the 75% threshold. **(D)** Dot plot of canonical markers for B cells, monocytes, T cells, natural killer cells, dendritic cells, and platelets. The dot size represents the gene (x-axis) percent expression on its corresponding cluster (y-axis). The color represents the average expression of the genes (gray: low, red: high).

To discover the SLE-dominated alterations of PBMC composition in SLE patients, two single-cell transcriptomic datasets with more than 3.15 × 10^5^ cells from 15 SLE patient (GSE137029) and 8 healthy donor (GSE164378) samples were enrolled in our study. The uniform manifold approximation and projection (UMAP) and Louvain algorithm were applied for unsupervised dimension reduction and clustering, respectively (32, 33). As shown in **Figures 2A, B**, the PBMCs of these two datasets could be grouped into sixteen molecularly distinct clusters. The clusters were annotated based on the gene expression values compared to all other cells. The results illustrated two clusters of T cells, B cells, natural killer cells, and erythroid cells, three clusters of monocytes and dendritic cells, and one platelet cluster (**Figures 2A, D** **)**. Unfortunately, SLE-dominated (clusters 13 and 15) clusters were tiny and might come from erythrocytes (HBB specifically expressed). The rest of the cell cluster proportions of SLE patients and healthy donors were evenly balanced or healthy donor dominant (**Figure 2C**). This is partly because the difference between SLE patients and healthy donors might be attenuated under the overall PBMC perspective. Hence, to strengthen the power of detecting SLE-dominant information, further analyses were performed in the subpopulations of PBMCs according to the cluster annotation above.

### Identification of SLE-Dominated Clusters in B Cells and Monocytes

**Figure 3:**
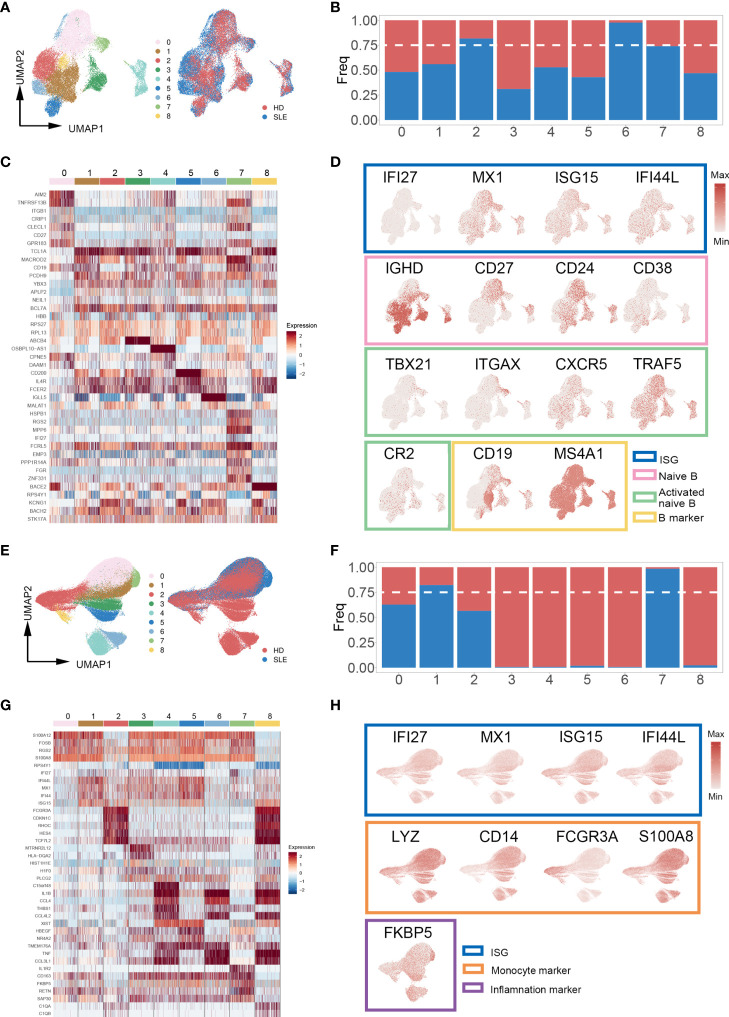
Cell proportion analysis of re-clustered B cells and monocytes. **(A, E)** UMAP plot of re-clustered B cells and monocytes from SLE patients and healthy donors, respectively. Left panel cells were categorized with Louvain clusters; the right panel cells were categorized by their source (SLE patient/healthy donors). **(B, F)** Bar plot of cell proportion in each B cell and monocyte subcluster, respectively. The dashed line represents the 75% cell proportion threshold. Both B cells (clusters 2, 6, 7) and monocytes (clusters 1, 7) have a unique cell subpopulation where SLE is predominant. **(C, G)** Heatmap of top five cluster-specific genes of each subclusters within B cells and monocytes, respectively. The color represents the expression level (blue: low, red: high). **(D, H)** UMAP plot of selected gene expression in re-clustered B cells and monocytes, respectively.

Increasing evidence indicates that specialized immune cell subsets are involved in the pathophysiological process of autoimmune diseases through multiplex pathways and signals (34–36). Thus, we re-clustered the subpopulations of PBMCs to identify the SLE-dominated clusters in which the cell proportion of SLE exceeds 75%. Interestingly, the SLE-dominated clusters were identified only in B cells (clusters 2, 6, and 7, **Figures 3A, B** **)** and monocytes (clusters 1 and 7, **Figures 3E, F** **)**; the rest of the PBMC subpopulation is shown in **Figure S2**. With differential expression gene (DEG) analysis on B cells and monocytes, the top five cluster-specific genes based on their log2 fold change values are shown in **Figures 3C, G**, respectively. All DEG analysis results are shown in **Table S1**. Interferon inflammatory signatures are closely related to the SLE (37). Consistently, we found that cluster 7 of B cells has interferon-stimulated gene (ISG) expression patterns (IFI27, MX1, ISG15, and IFI44L). Moreover, we identified that this cluster simultaneously possess the typical expression patterns of naïve and autoactive B lymphocytes (naïve: IgD+, CD27-, CD38 low, CD24 low; autoactive: TBX21, ITGAX, CXCR5, TRAF5, CR2, **Figure 3D**) (38, 39). In addition, we also found that cluster 1 of monocyte highly expressed ISGs (IFI27, MX1, ISG15, IFI44L), and cluster 7 of monocyte had a proinflammatory character (FKBP5, **Figure 3H**) (40).

Taken together, these findings revealed that there were enhanced signals of an autoreactive/inflammatory state in B cells and monocytes of SLE patients, which suggested the essential roles in the pathophysiological process of SLE.

### Weakened Interactions Among the PBMC Subpopulations of SLE Patients

To systematically explore the alterations of PBMCs in SLE patients and obtain a powerful source of information for the training of the machine learning model, we employed CellChat to analyze cellular cross talking from scRNA-seq data. Three scRNA-seq datasets (GSE137029, 15 adult patients with SLE; GSE135779, 13 child patients with SLE; GSE164378, 8 healthy donors) with more than 3.80 × 10^5^ cells were included in this analysis.

**Figure 4:**
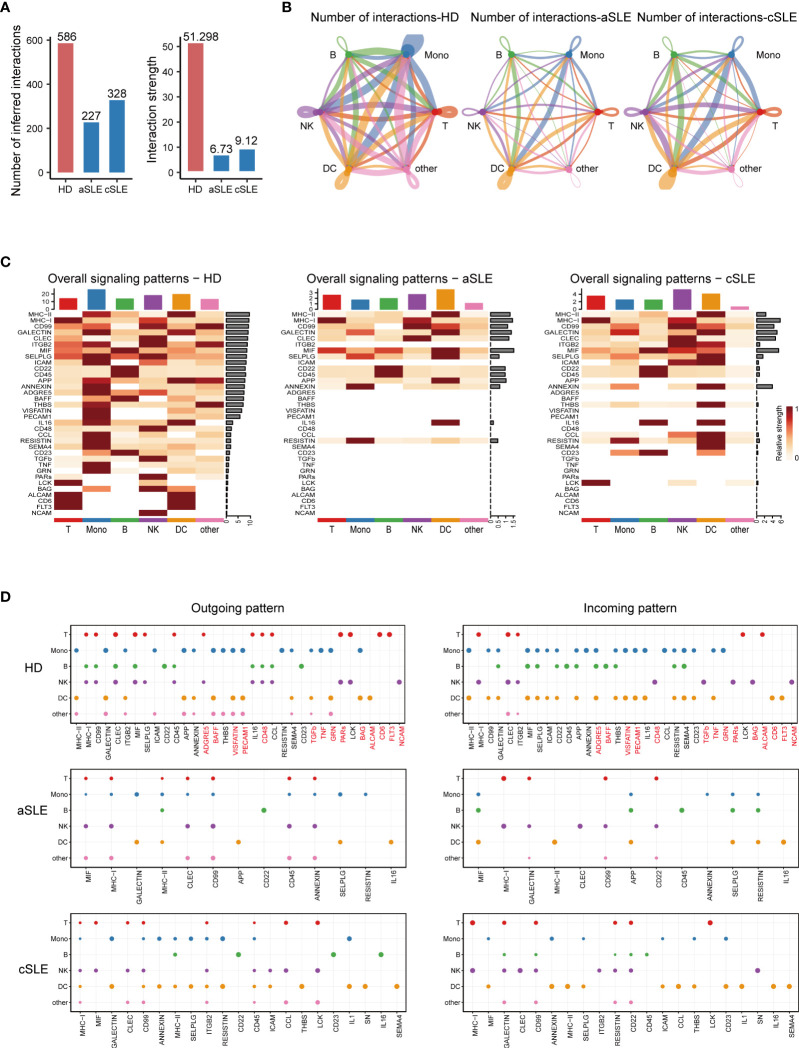
CellChat analysis of whole PBMCs from SLE patients and healthy donors. **(A)** Bar plot of the overall difference among healthy donors (HD), adult SLE patients (aSLE), and child SLE patients (cSLE). The left panel shows the total number of interactions, and the right panel shows the interaction strength. **(B)** Circle plot of PBMC subpopulation among HD, aSLE, and cSLE. The line width: the connection strength; dark blue: monocytes, green: B cells, red: T cells, purple: natural killer cells, orange: dendritic cells and pink: other cells. These together revealed a weakened PBMC subpopulation cross talking and distinct signal pattern under SLE. **(C)** Heatmap reveals the overall signal pattern changes in the HD, aSLE, and cSLE groups, and the signal strength is scaled from white (no signal detected) to dark red (strong). **(D)** Dot plot for the emergence probability of signal outgoing (left panel) and incoming (right panel) patterns within each PBMC subpopulations among HD, aSLE, and cSLE. The dot size represents the *p* value. Patterns which specifically disappeared under disease state were marked with red. The total number of outgoing and incoming signal reduced significantly in SLE.

The total number and strength of ligand–receptor pairs were significantly reduced in both adult and child SLE patients compared with healthy donors (**Figure 4A**). Remarkably, the interactions of PBMC subpopulations in SLE patients were weakened (**Figure 4B**). Comparing overall and detailed outgoing/incoming signaling pattern variations among SLE and healthy donors, we identified that abundant signal patterns could be observed for the healthy donors, but in the SLE groups, the number of involved pathways was reduced (**Figures 4C, D** **)**. In detail, there were several signal patterns that specifically disappeared under the disease state. Among them, FLT3, CD48, and TGF-beta signal patterns have been reported to have a negative correlation with SLE development (41–44). Taken together, the disappearance of multiple signal patterns might be a potential feature during SLE development.

### Detailed Ligand-Receptor Pair Alterations in SLE Patients

**Figure 5:**
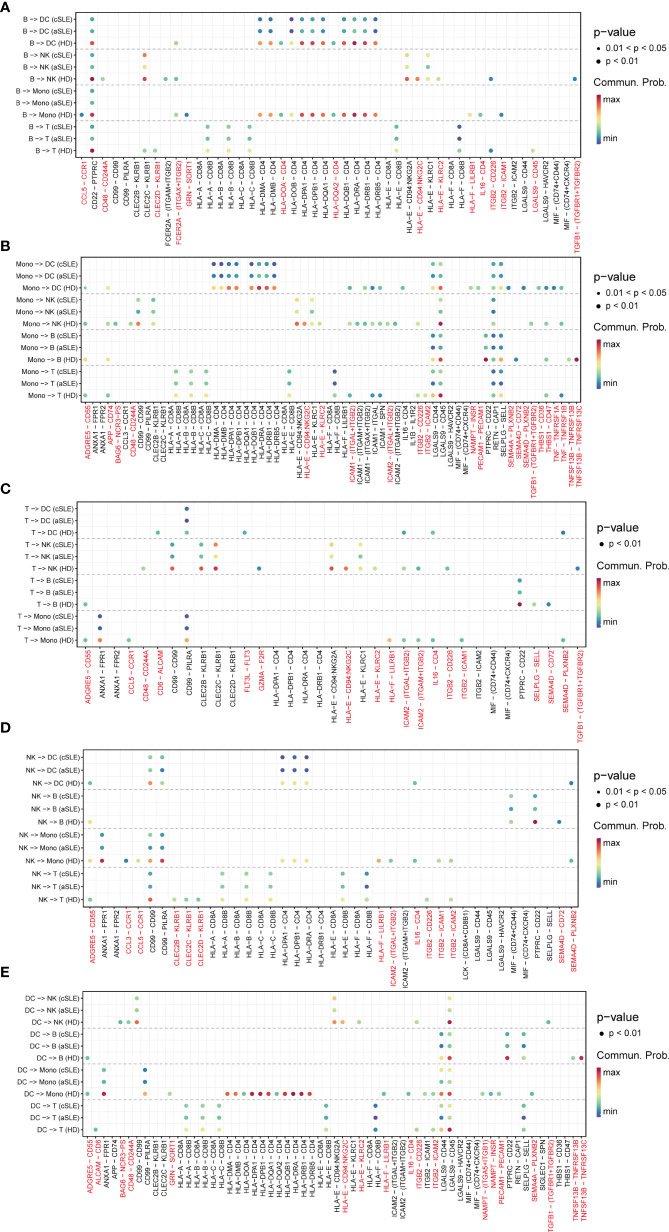
Ligand–receptor pair alternation of SLE patients compared with healthy donors. Dot plot for the emergence probability of ligand–receptor pairs within each PBMC subpopulations **(A)** B cells, **(B)**monocytes, **(C)** T cells, **(D)** natural killer cells, **(E)** dendritic cells) among HD, aSLE, and cSLE. The dot color represents the probability. Pairs which specifically disappeared under disease state are marked with red.

As the above results indicated that numerous signal patterns disappeared in SLE compared with healthy states, to find detailed information, we further explore the discrepancy of ligand–receptor pairs from all PBMC subpopulations (B cells, monocytes, T cells, natural killer cells, and dendritic cells) among healthy donor, adult SLE (aSLE), and child SLE (cSLE) groups (**Figures 5A–E**). We identified that eighty-seven ligand–receptor pairs disappeared in SLE patients, which were composed of sixty-one genes. The frequency of each gene appeared at each PBMC subpopulation, as listed in **Table S2**. The genes which showed more than two kinds of PBMC subpopulation were recognized as significant ones to be selected as a second part of feature input for machine learning.

Among them, TGFBR1, TGFBR2, CCL5, CD48, CD244A, and CD72 have been reported to be closely related to the pathophysiologic processes of autoimmune diseases (41, 43, 45–47). For example, TGFBR1, TGFBR2, and CCL5 levels are negatively correlated with SLE development (43, 45). CD48, also known as SLAMF2, which could regulate both natural killer cells and cytotoxic CD8+ T cells (48), could protect mice from autoimmune nephritis (41), CD244A and CD72 were specifically decreased in monocytes and B cells during SLE development (47, 49). Interestingly, all these selected pairs are all in B cells or monocytes, suggesting the key roles of monocytes and B cells on the pathophysiologic processes of autoimmune diseases. All these findings were consistent with our results of integration analysis.

### Efficient Machine Learning Models for Chronic Autoimmune Disease Diagnosis

To establish a mathematical model of unbiased random forest machine learning for SLE accurate diagnosis, sixty-seven top five cluster-specific genes derived from integration analysis and twenty-one significant genes identified *via* cellular cross-talking analysis were combined as feature input. The dataset GSE135779, containing 3.60 × 10^5^ PBMCs (derived from 33 cSLE, 7 aSLE, and 11 healthy children, 5 healthy adults), was included to evaluate the diagnosis efficiency of our mathematical model.

**Figure 6:**
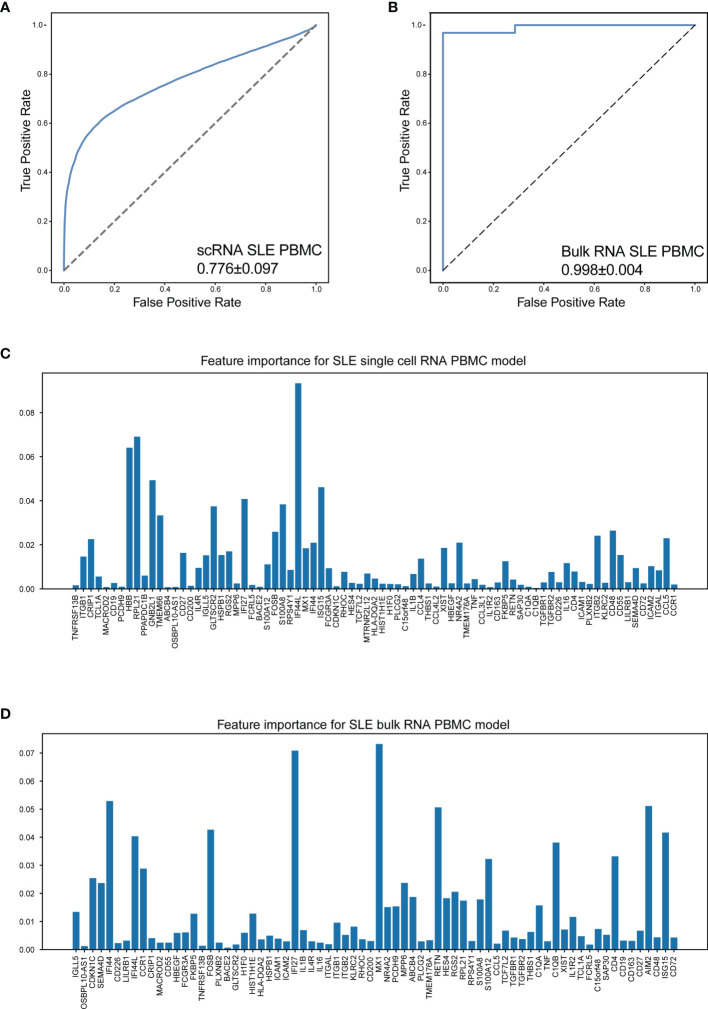
Machine learning model accurately distinguish SLE. **(A)** The performance of distinguish SLE using scRNA-seq data of PBMCs (AUC = 0.776 ± 0.097). **(B)** The performance of distinguish SLE using bulk RNA-seq data of PBMCs (AUC = 0.998 ± 0.004). **(C, D)** Bar plot for the corresponding feature importance within the correlated model using scRNA-seq and bulk RNA-seq data, respectively. The bar length: feature importance.

The results indicated that our machine learning model could separate SLE and healthy status with acceptable accuracy (AUC = 0.776 ± 0.097, **Figure 6A**). The feature importance of our gene set for SLE is shown in **Figure 6C**. Considering the signal intensity of our gene sets and the denoising ability of machine learning, a further investigation was conducted to evaluate the disease distinguishing the efficiency of our mathematical model using bulk RNA-seq data. The bulk RNA-seq datasets (GSE72509, GSE183204), which include 99 SLE patients and 30 healthy donors were used in this investigation. The results indicated that our mathematical model has great adaptability (AUC = 0.998 ± 0.004, **Figure 6B**). The corresponding feature importance was also calculated (**Figure 6D**). This revealed that combined with the unbiased random forestry machine learning model, our gene sets rendered a powerful mathematical tool for distinguishing SLE.

It is reported that chronic autoimmune diseases including SLE and RA might share some similar cellular pathogeneses with MS (50). Thus, we investigated whether our machine learning model could efficiently distinguish RA and MS based on bulk RNA-seq data. Three datasets were included in this study, including a set of PBMC datasets (GSE90081, GSE183204) with 12 RA patients and 24 healthy donors, a synovial tissue dataset (GSE89408) with 152 RA patients and 28 healthy donors, and a PBMC dataset (GSE159225) with 20 relapse-and-remission MS patients, 10 secondary progressive MS patients, and 20 healthy donors.

**Figure 7:**
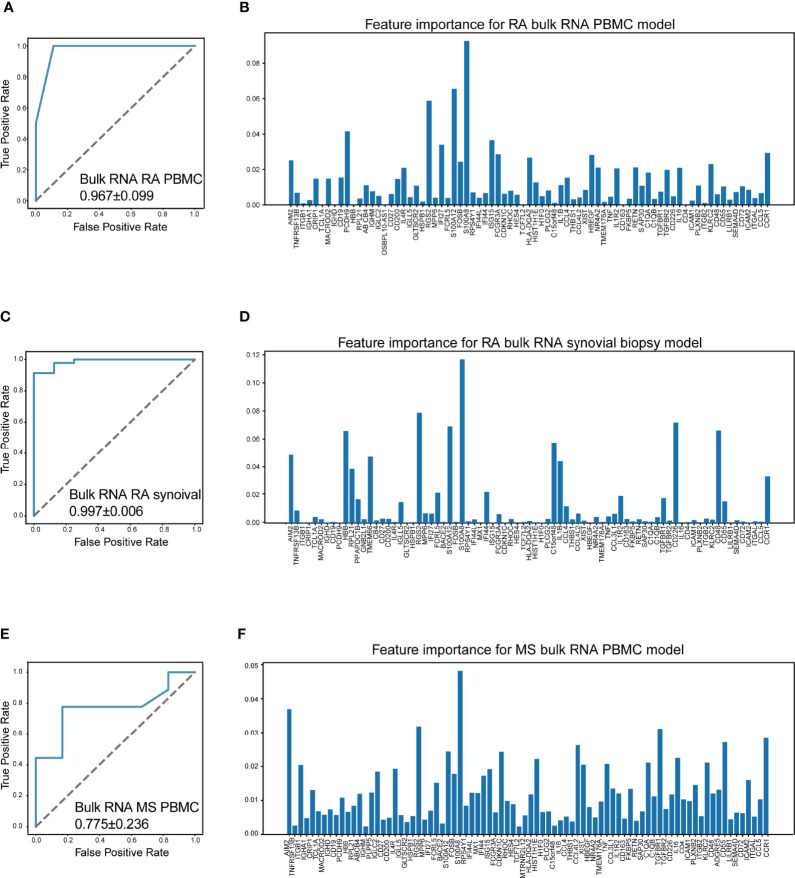
Machine learning model accurately distinguish RA and MS. **(A)** The performance of distinguish RA (rheumatoid arthritis) using bulk RNA-seq data of PBMCs (AUC = 0.967 ± 0.099). **(B)** Bar plot for the feature importance with the correlated model. **(C)** The performance of distinguish RA using bulk RNA-seq data of synovial tissue (AUC = 0.997 ± 0.006). **(D)** Bar plot for the feature importance with the correlated model. **(E)** The performance of distinguish MS (multiple sclerosis) using bulk RNA-seq data of PBMCs (AUC = 0.775 ± 0.236). **(F)** Bar plot for the feature importance with the correlated model. The bar length: feature importance.

Surprisingly, our machine learning model could separate patients with RA/MS and healthy donors with excellent accuracy in RA patients (AUC = 0.967 ± 0.099 in RA PBMC datasets, **Figure 7A**; AUC = 0.997 ± 0.006 in the RA synovial dataset, **Figure 7C**). For MS patients, our figure rendered an acceptable accuracy (AUC = 0.775 ± 0.236 in MS PBMC datasets, **Figure 7E**). The corresponding feature importance shown in **Figures 7B, D, F** illustrated that although our gene sets have extensive applicability and great accuracy for these diseases, each gene has different importance across each of these diseases. It suggested that our machine learning model requires a fine adjustment when applied to these diseases.

To determine the contribution of positive signals to the accuracy of our machine learning model, we obtain a public bulk RNA-seq dataset (GSE137143, 122 MS patients and 22 healthy donors), which consists of only CD14-positive monocytes. Unfortunately, the AUC value dropped to 0.673 ± 0.136, indicating that the accuracy sharply decreased (**Figure S4**). This result suggested that the distinguishing power of our model was reduced on account of a loss of positive signals, for example, the signals from B cells.

### Diagnostic Accuracy Validation of the Machine Learning Model

**Figure 8:**
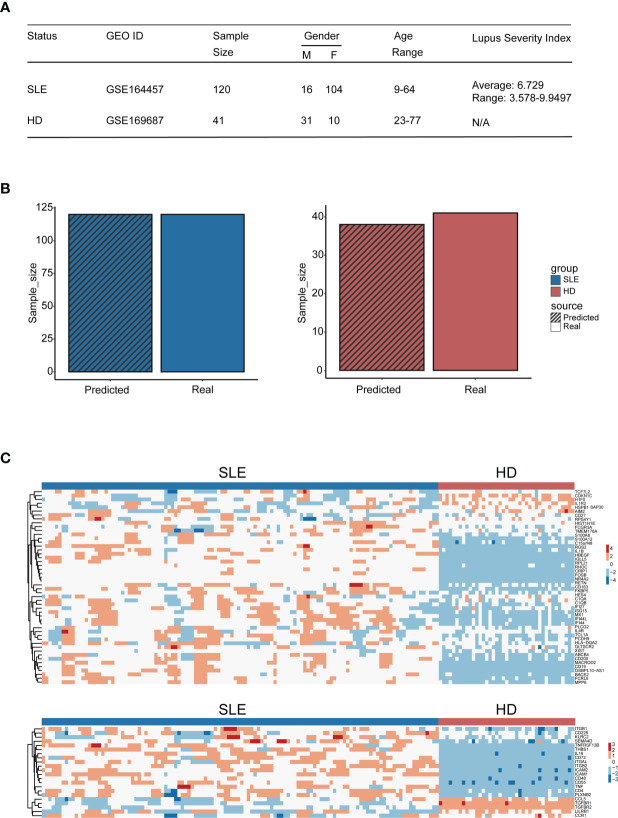
Diagnostic accuracy validation of the machine learning model. **(A)** Table of cohort basic information. **(B)** Bar plot of the amount of SLE patients and healthy donors being distinguished accurately by the model (blue: SLE patients, red: HD); the bar with black stripe represents the model-predicted number, while the other represents the real number. **(C)** Heatmap of genes used for machine learning setup within the validation cohort (the upper panel: genes derived from the differential expression gene identification within integration analysis, the lower panel: genes derived from CellChat analysis).

To evaluate the diagnosis accuracy of our machine learning model, an independent cohort containing 120 SLE patients (GSE164457) and 41 healthy donors (derived from GSE169687) were enrolled into the study. The basic information and the gene expression pattern of objects within this cohort are shown in **Figures 8A, C**. Notably, the precision rate of our machine learning model diagnosis was 100% (120/120) and 92.7% (38/41) for SLE patients and healthy donors, respectively (**Figure 8B**). This result confirmed the diagnostic accuracy of our machine learning model, which suggested that it may be feasible to develop into an efficient tool for accurate disease diagnosis in the future.

## Discussion

We aimed to develop a feasible strategy for distinguishing patients with SLE and other major chronic autoimmune diseases in the early stage from healthy people. To achieve our purpose, the most crucial information that characterizes diseases should be filtered out first. From public single-cell RNA sequencing datasets, we found that B cells and monocytes were the only two subpopulations containing SLE-dominated clusters in the PBMCs of patients, which suggested that they might carry much stronger signals that indicate SLE than other PBMC subpopulations. To date, conclusions about the contribution of PBMC subpopulations to the development of SLE and other autoimmune diseases are not consistent, even when based on single-cell RNA sequencing data (51–55). Most studies mainly focus on specific disease aspects, which might result in imbalanced data selection, background noise interference, and biased conclusions. Hence, we selected the single-cell RNA sequencing data from over 1.50 × 10^5^ cells for each category with a balanced ratio between patients and controls (approximately 1:1) to avoid rushing into any prejudicial conclusions.

Further investigation of differentially expressed genes revealed the details of the most significant information that marks a disease within B cells and monocytes. A few interferon-stimulated genes were active in the SLE-dominated B cells and monocytes, indicating that these cells might be a consequence of the inflammatory microenvironment. It is well known that the inflammatory microenvironment may be crucial to the progression of SLE and other chronic autoimmune diseases. Tsokos et al. reported that the production of autoantibodies triggered by both the innate and adaptive immune responses against self-antigens in SLE patients resulted in the accumulation of monocytes and activation of lymphocytes (56). Our results confirmed this suggestion. Interestingly, we found an activated naïve cluster of B cells in the SLE-dominated clusters. Recently, Jenks et al. reported a distinctive differentiation fate of autoreactive naïve B cells (39). This was similar to our finding and suggested that B cells should play an important role in the development of SLE.

All of the PBMC subpopulations were influenced mutually in the progression of chronic autoimmune diseases, and analyses based on individual subpopulations may lose important information of reciprocities that accounts for disease progression. Most current scRNA-seq data analysis tools focus on detailed categorizations and trajectories of cells (28, 57–59). Recently, bioinformatic tools (e.g., CellChat, CellPhoneDB, iTALK) were developed to infer cellular cross talking from scRNA-seq data, which make it possible to decipher reciprocities among cells under a single-cell level (57, 60–62). Therefore, we carried out cellular cross-talking analyses to reveal dynamic interactions across PBMC subpopulations and systematically decipher the etiology of diseases. Surprisingly, we found that the interactions among the PBMC subpopulations of SLE patients were weakened. It was reported that monocytes might contribute to the hyperactivity of B cells in SLE patients (63). A study also revealed that monocytes may function as a bridge during RA pathogenesis, and colocalization of CD14+ cells with CD4+ T effectors was found at sites of the inflamed rheumatoid synovium (64). Together, these reports illustrate that immune cells weave a network and that their interaction would provide significant information for autoimmune disease pathogenesis. Further detailed analysis revealed that the major changes occurred in B cells or monocytes, including FLT3, CD48, TNF, and TGF-beta signal patterns that have been reported to have a negative correlation with SLE development (41–44). Our results were consistent with previous studies on the variations in B cells (65–67) and monocytes (68–70) in SLE. Considering the repeatable results gained from our study, it should be convincing that the interactions among the PBMC subpopulations of SLE patients may be weakened, which could result in abnormal emergences or variations in signaling patterns within PBMCs.

Based on our finding of powerful information that characterizes diseases, we tried to establish a machine learning model to distinguish chronic autoimmune diseases. Several reports have proven that the random forest (RF) machine learning method would give a high accuracy in disease classification when abundant features were included (71, 72), and another reason for the random forest model was its interpretability—each gene contribution in the RF machine learning model was visible. Our area under curve (AUC) score for SLE indicates that our machine learning model has the potential to become an efficient tool for accurate diagnosis of SLE at the single-cell RNA level. Considering that the information we identified was not specific to the early stage of the disease, further optimization should be performed to identify the sensitive information in the early stage of the disease to strengthen the diagnostic power of our machine learning model.

Further investigation is also needed to evaluate the efficiency of our machine learning model using bulk RNA-sequencing data. Our AUC score illustrates that although other immune cell background noise might be introduced into RNA-seq data, the gene set still has high accuracy in distinguishing patients with the disease from healthy donors. This might be attributed to the low correlation between each gene since they were derived from the two different analysis frameworks, and this low gene correlation in turn increased the random forest model accuracy (73). Given the cost and convenience of bulk RNA sequencing, our results suggested that this machine learning model should be highly applicable going forward. In addition, our classification results for bulk RNA sequencing data of PBMCs and synovial tissues derived from RA and MS patients indicated that this machine learning model also showed high accuracy in distinguishing these diseases. Numerous studies have reported that chronic autoimmune diseases, such as SLE, RA, and MS, might share some similar cellular pathogeneses (46, 50, 74). Our findings further confirmed this viewpoint and suggested that this machine learning model with the information we filtered out might be powerful enough to discriminate patients with common chronic autoimmune diseases from healthy donors, not just SLE patients.

## Data Availability Statement

The datasets presented in this study can be found in online repositories. The names of the repository/repositories and accession number(s) can be found in the article/**Supplementary Material**.

## Author Contributions

Author contributions are shown as follows. Conception and design: AX, YM, and WH. Acquisition of data: XX, YQ. Analysis and interpretation of data: TW, LZ, JC and YM. Writing, review, and/or revision of the manuscript: all authors. All authors contributed to the article and approved the submitted version.

## Funding

This work was supported by the National Natural Science Foundation of China (81730005), the National Key Research and Development Program of China, Stem Cell and Translational Research (2018YFA0107203), the National Natural Science Foundation of China (32130046) and the National Natural Science Foundation of China (81970222) and the Key Scientific and Technological Projects of Guangdong Province (2016B0-90918040, 2017B020230004).

## Conflict of Interest

The authors declare that the research was conducted in the absence of any commercial or financial relationships that could be construed as a potential conflict of interest.

## Publisher’s Note

All claims expressed in this article are solely those of the authors and do not necessarily represent those of their affiliated organizations, or those of the publisher, the editors and the reviewers. Any product that may be evaluated in this article, or claim that may be made by its manufacturer, is not guaranteed or endorsed by the publisher.
