# Supplementary figures and images for: Accurate Machine Learning Model to Diagnose Chronic Autoimmune Diseases Utilizing Information From B Cells and Monocytes
Source: Front Immunol. 2022 Apr 20;13:870531. doi: 10.3389/fimmu.2022.870531 (PMC9065417; doi:10.3389/fimmu.2022.870531)

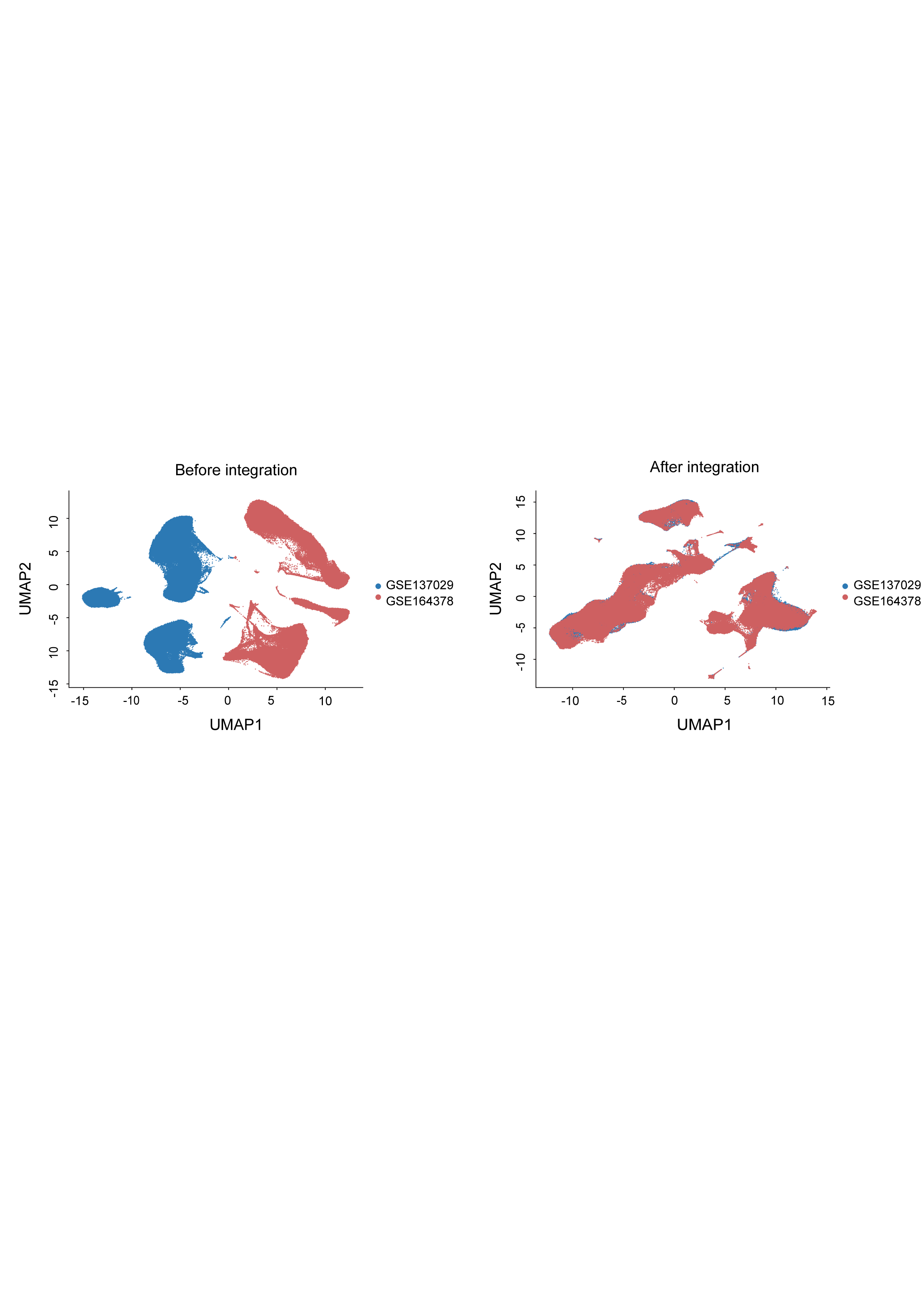

Supplement: Supplementary file 1 [file Image_1.tif]

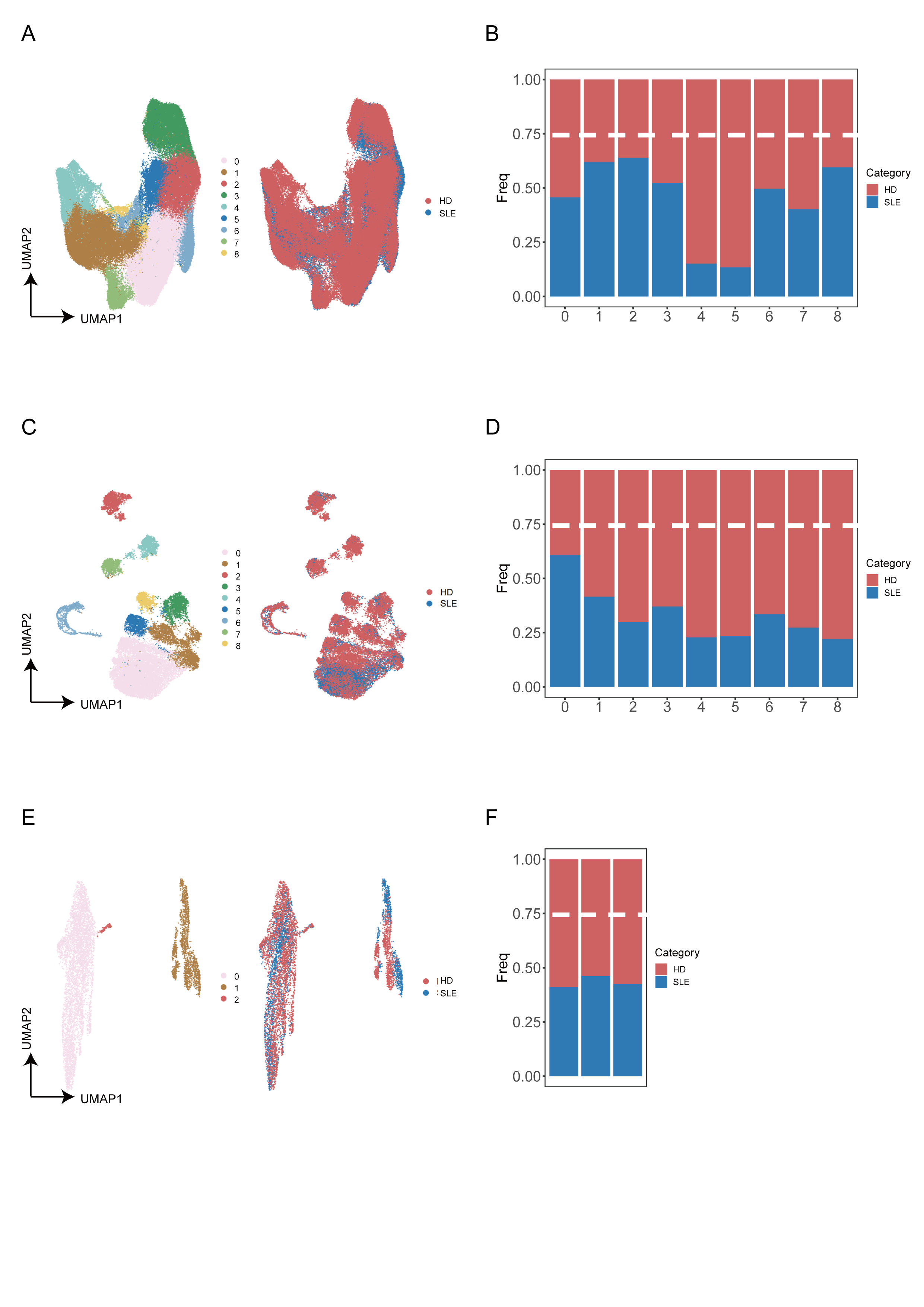

Supplement: Supplementary file 2 [file Image_2.tif]

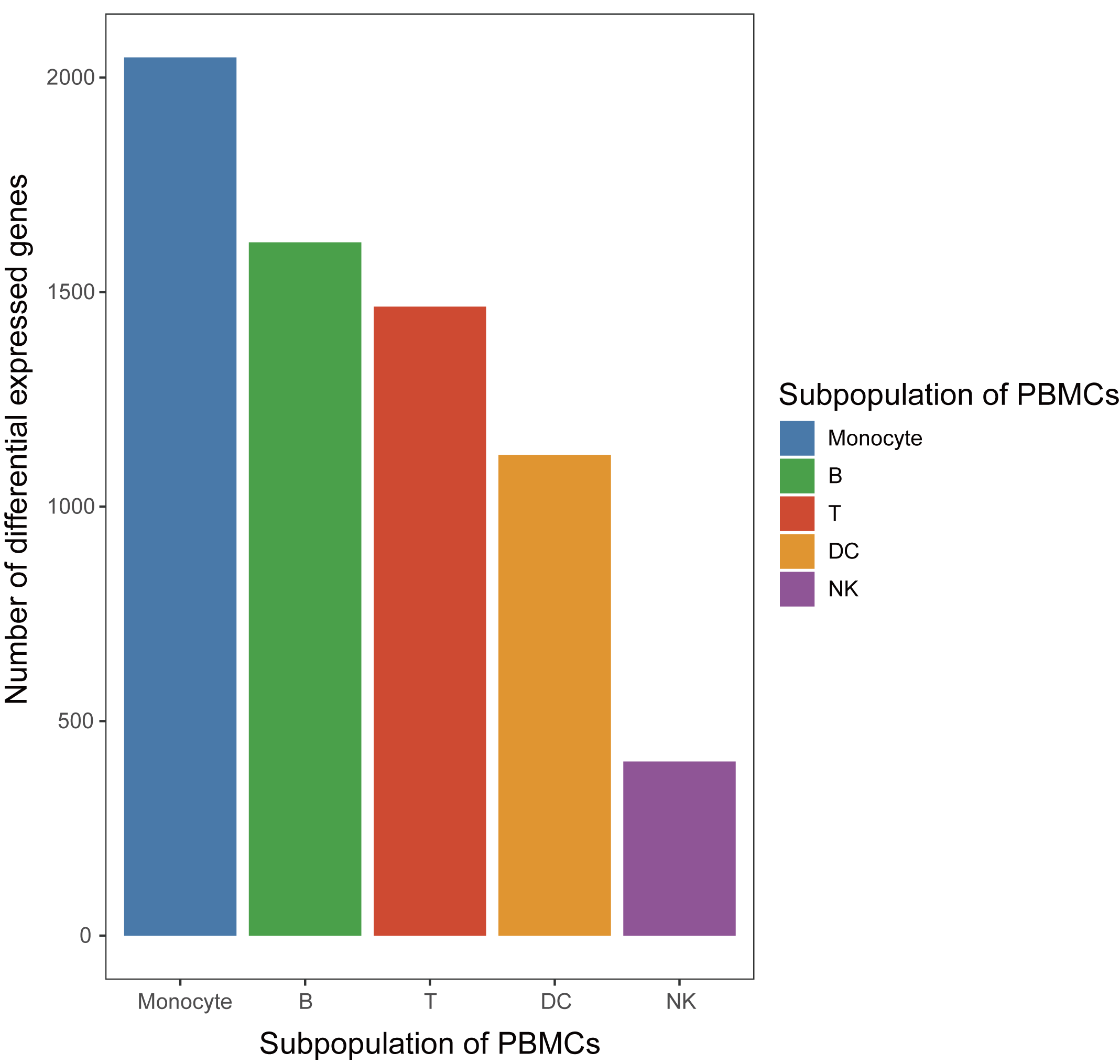

Supplement: Supplementary file 3 [file Image_3.tif]

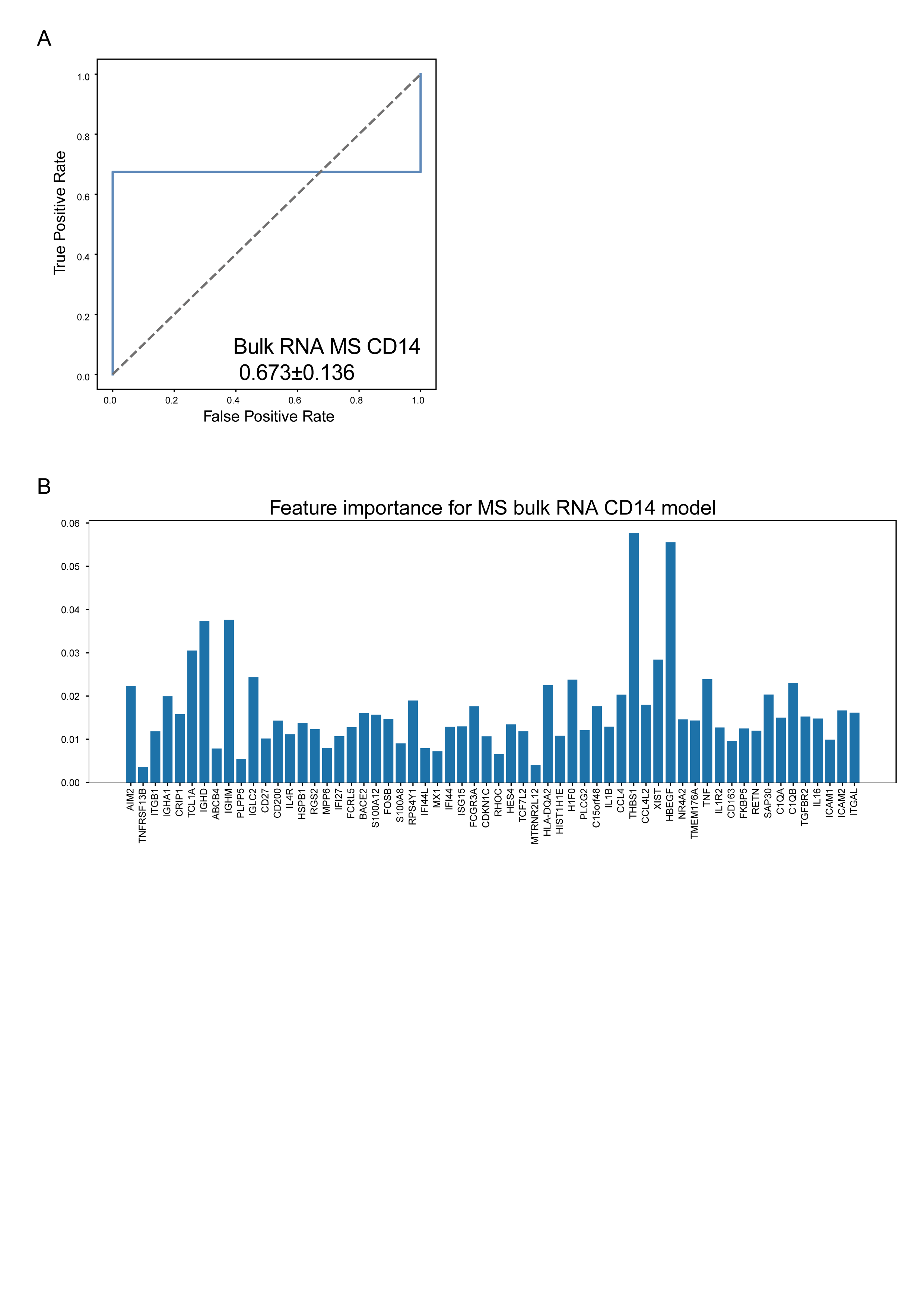

Supplement: Supplementary file 4 [file Image_4.tif]
